# Electrically Tunable Bifocal Metalens with Diffraction‐Limited Focusing and Imaging at Visible Wavelengths

**DOI:** 10.1002/advs.202102646

**Published:** 2021-09-05

**Authors:** Trevon Badloe, Inki Kim, Yeseul Kim, Joohoon Kim, Junsuk Rho

**Affiliations:** ^1^ Department of Mechanical Engineering Pohang University of Science and Technology (POSTECH) Pohang 37673 Republic of Korea; ^2^ Department of Biophysics Institute of Quantum Biophysics Sungkyunkwan University Suwon 16419 Republic of Korea; ^3^ Department of Chemical Engineering Pohang University of Science and Technology (POSTECH) Pohang 37673 Republic of Korea; ^4^ POSCO‐POSTECH‐RIST Convergence Research Center for Flat Optics and Metaphotonics Pohang 37673 Republic of Korea; ^5^ National Institute of Nanomaterials Technology (NINT) Pohang 37673 Republic of Korea

**Keywords:** bifocal metalens, liquid crystals, switchable devices, tunable bifocal metalens

## Abstract

Tunable optical devices powered by metasurfaces provide a new path for functional planar optics. In particular, lenses with tunable focal lengths can play a key role in various fields with applications in imaging, displays, and augmented and virtual reality devices. Here, the authors demonstrate an electrically controllable bifocal metalens at visible wavelengths by incorporating a metasurface designed to focus light at two different focal lengths, with liquid crystals to actively manipulate the focal length of the metalens through the application of an external bias. By utilizing hydrogenated amorphous silicon that is optimized to provide an extremely low extinction coefficient in the visible regime, the metalens is highly efficient with measured focusing efficiencies of around 44%. They numerically design and experimentally realize and characterize tunable focusing and demonstrate electrically tunable active imaging at visible wavelengths using the bifocal metalens combined with liquid crystals. Diffraction limited focusing and imaging is verified through the analysis of the measured optical intensities at the focal points and the modulation transfer function. The bifocal metalens is used to demonstrate electrically modulated focus switching between the two designed focal planes, to display images of positive and negative target objects.

## Graphical abstract

An electrically tunable bifocal metalens is designed and experimentally realized for diffraction limited focusing and imaging at visible wavelengths. By combining propagation and geometric phase, the metalens produces a measured efficiency of around 44%.

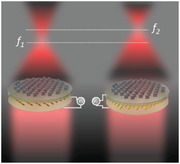

## 1 Introduction

Metasurfaces consisting of arrays of subwavelength nanostructures, known as meta‐atoms, have been introduced as an alternative to conventional optical elements, with various applications such as imaging,^[^ ^1^ ^]^ structural coloration,^[^ ^2^, ^3^, ^4^, ^5^ ^]^ and holography.^[^ ^6^, ^7^, ^8^ ^]^ Their planar nature has opened the door for the miniaturization of optical devices, without having to considerably compromise the performance. Recently, there has been a great deal of development in the field of metalenses.^[^ ^9^, ^10^, ^11^, ^12^, ^13^, ^14^ ^]^ These planar lenses utilize the power of metasurfaces by manipulating the phase of incident light at the subwavelength scale to produce diffraction limited focusing.^[^ ^15^ ^]^ All of the same considerations that exist in conventional optics are still relevant for metalenses, such as the various aberrations, size limitations, and the working bandwidth. A number of high‐performance metalenses have been proposed and experimentally demonstrated to overcome these difficulties. Broadband,^[^ ^16^, ^17^ ^]^ achromatic,^[^ ^18^, ^19^ ^]^ and high numerical aperture^[^ ^20^, ^21^ ^]^ (NA) metalenses have paved the way for a new era of thin, planar lenses that have significantly smaller footprints than their conventional glass‐based cousins. This miniaturization of lenses could have a remarkable influence on all kinds of optical applications, from lab‐based microscopy to consumer grade devices.

Varifocal optical elements are extremely important for focusing and imaging in three dimensions. The control of the focal length in conventional optics is often achieved through mechanically moveable, high‐precision glass elements that are heavy and bulky. In contrast, varifocal metalenses are naturally small and lightweight giving them clear advantages in many situations. However, although the modulation of the optical properties of metasurfaces through tuning at the meta‐atom level has been demonstrated at longer wavelengths,^[^ ^22^ ^]^ it is extremely difficult for applications in the visible regime due to the requirement of subwavelength sized meta‐atoms that would require extremely precise modulation. Therefore, most demonstrations of varifocal metalenses so far have relied on a method of tuning the properties of the metalens as a whole. These have come in the form of micro‐electromechanical systems (MEMS),^[^ ^23^ ^]^ actuating lens doublets,^[^ ^24^ ^]^ stretchable substrates,^[^ ^25^, ^26^ ^]^ and through the use of phase change materials (PCMs).^[^ ^27^ ^]^ The first three methods rely on physically moving parts, which could be suspect to degradation over time, and require precise control over extremely small distances to function at visible wavelengths. PCMs are an exciting option for metasurfaces, however their refractive index modulation and significant optical losses at visible wavelengths are still hurdles that need to be overcome. The electrical modulation of the focal length of metalenses has also been proven using graphene to tune the Dirac point,^[^ ^28^ ^]^ and through the integration of liquid crystals (LCs).^[^ ^29^ ^]^ A comparison of the reported work on varifocal metalenses, including the operating wavelengths, tuning speed, and focal length range is provided in Note S1, Supporting Information.

The integration of LCs with metasurfaces has been proven as an excellent opportunity for creating tunable devices based on metasurfaces,^[^ ^30^, ^31^, ^32^, ^33^, ^34^ ^]^ and has already been exploited to demonstrate chromatic aberration control of a metalens.^[^ ^35^ ^]^ The introduction of LCs into metasurfaces provides a new degree of freedom that can be controlled at will through the application of an external stimulus. The optical modulation from the LCs can be designed specifically for the purpose, for example, to control the polarization of the light incident on the metasurface, or the refractive index of the medium surrounding it. Therefore, the type of LC should be chosen specifically to suit the needs of the device. Furthermore, not only can LCs be controlled electrically, but also thermally, and through the application of external pressure. These multiple operating methods open up new avenues towards completely active devices with extended functionalities.

**Figure 1:**
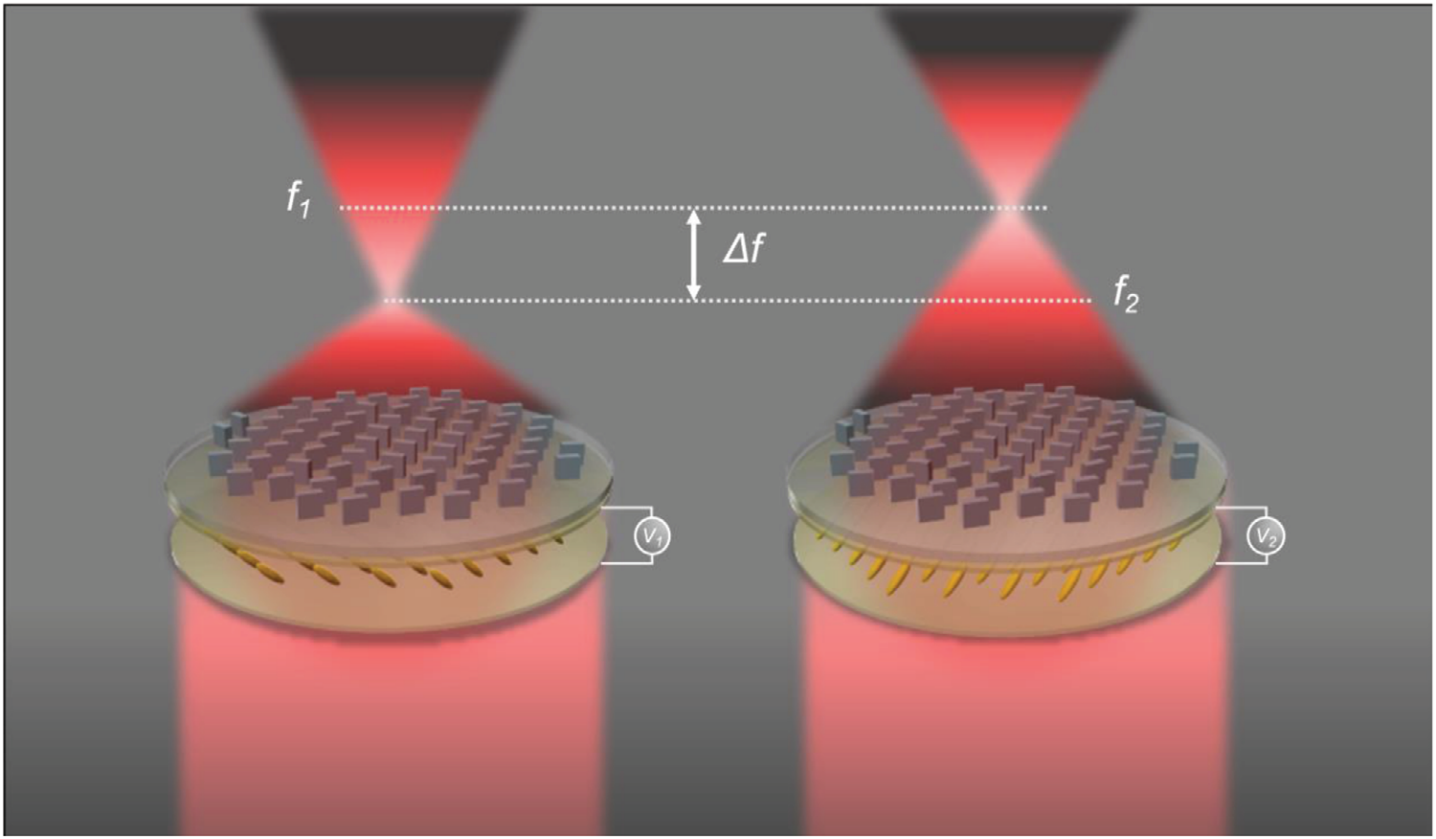
Schematic illustration of the bifocal metalens. By applying an external bias (from *V*_1_ to *V*_2_) to the LC cell, the orientation of the LCs causes a change between RCP and LCP light which in turn produces a change in focal length from *f*_1_ (7.5 mm) to *f*_2_ (3.7 mm).

Here, we demonstrate a bifocal metalens that incorporates a LC cell used to control the polarization of the incident light for tunable focusing and imaging. Specifically, by applying an electrical bias to the LC cell, the polarization of the incident light is modulated between right circularly polarized (RCP) to left circularly polarized (LCP) which causes the focal length of the metalens to change between two chosen locations. A schematic diagram of the device is shown in **Figure** **1**. By arduously designing the metalens to include the phase profiles for lensing at two different focal lengths for the two incident polarization states and integrating it with an LC cell, we numerically design and experimentally demonstrate an actively tunable bifocal metalens for focusing and imaging at visible wavelengths. This work demonstrates that the integration of LCs with metalenses can lead to advanced functionalities that could have profound impacts on optical systems that require actively tunable responses, beyond static metalenses. Furthermore, compared to a recently published experimental example of an electrically tunable bifocal metalens using liquid crystals^[^ ^36^ ^]^ to modulate the effective local refractive index around the metalens, imparting some form of extra phase delay to produce a difference in focal point, our approach is to use the LCs in a way to control the polarization of the incident light. This design strategy allows us to use a much simpler meta‐atom design process, avoiding the arduous calculation and selection of suitable meta‐atoms from a huge library of geometric parameters, and also proves better performance in terms of focusing efficiency. Moreover, we experimentally demonstrate electrically tunable imaging of negative and positive targets.

## 2 Results and Discussion

The interactions of light and subwavelength sized meta‐atoms have been investigated deeply. It is well‐known that through the careful design of the dimensions of the meta‐atoms and correct selection of material, the phase, amplitude, and polarization of light can be locally controlled in order to produce any arbitrary optical properties. For the control of phase, two approaches, namely propagation and geometric phase, have been actively utilized. Propagation phase, also known as retardation phase, allows for independent phase profiles for two orthogonal linearly polarized states of light. This is achieved through the careful design of the meta‐atoms, usually by adjusting the long and short axis of a rectangular shape. Since the dimensions of the meta‐atoms are small compared to the wavelength of incident light, we consider only the zeroth‐order propagation, as if from a plane wave. Then, the Jones matrix of a uniaxial crystal acting a phase plate, can bewritten as:

(1) $$J=R\left(-\theta \right)\left(\begin{array}{cc} e^{\left(i\phi_{x}\right)} & 0\\ 0 & e^{\left(i\phi_{y}\right)} \end{array}\right)R\left(\theta \right)$$

where *ϕ_x_* and *ϕ_y_* are the phase shifts for linearly polarized light along the fast and slow axes, rotated by an angle of *θ* relative to the reference coordinate system. *R* is a 2 × 2 rotation matrix given by:

(2) $$R\;\left(\theta \right)=\left(\begin{array}{cc} \cos\theta \; & \sin\theta \\ -\sin\theta \; & \cos\theta \end{array}\right)$$

By keeping the angle *θ* constant while changing the dimensions of the meta‐atoms, it is possible to create phase profiles for two orthogonal, linear polarization states, producing the well‐known propagation phase. Using this methodology, polarization dependent metasurfaces have been produced for holography^[^ ^37^ ^]^ and other applications. Metasurfaces based on the propagation phase have the inherent drawback that only half of the meta‐atoms are being utilized for a certain polarization state, meaning that the overall efficiency is capped at 50%. Meanwhile, geometric phase, also known as Pancharatnam–Berry (PB) phase, allows for the design of metasurfaces that produce phase profiles that are equal and opposite for RCP and LCP light. This phase manipulation stems from the polarization change of light as it passes through the meta‐atoms. It can be explained by understanding how two parts of a uniformly polarized wavefront can take different paths to reach the common output polarization state.^[^ ^38^ ^]^ The relative phase change that occurs between the two is then given by half of the solid angle enclosed by the paths. This is achieved using meta‐atoms that act as half‐wave plates, that is, by ensuring that |ϕ_x_ − *ϕ*_y_| *= π*, that are then rotated over the metasurface to enforce a phase profile of *ϕ*(*x*,*y*) = *2θ*(*x*,*y*) on one of the circularly polarized states of light, where *θ* is the angle that the meta‐atom is rotated at. It is clear to see that rotation angles of 0 to *π* can therefore produce PB phase that can be controlled linearly from 0 to 2*π*. The limitation of PB phase is that, due to symmetry, the phase profile that is produced for LCP light is the opposite for RCP, that is, *ϕ*_LCP_(*x*,*y*) = −*ϕ*_RCP_(*x*,*y*). Therefore, it is impossible to make a metalens that has two focal points in the same direction, as PB phase dictates that if the lens converges for LCP light, it would diverge for RCP light. However, these restrictions can be overcome by combining the two methods.^[^ ^39^, ^40^ ^]^ Here, we use this to design a bifocal metalens that has different focal lengths for the two states of circularly polarized light.

The required phase profile to focus an incident plane wave at a focal length (*f*) is given by:

(3) $$\psi \left(x,y\right)=-\;\frac{2\pi }{\lambda }(f-\sqrt{x^{2}+y^{2}+f^{2})}$$

where *x* and *y* are the positional coordinates of the metasurface, and *λ* is the wavelength of light. We define two phase profiles, *ψ*_*RCP*_(*x*,*y*) for RCP incident light, and *ψ*_*LCP*_(*x*,*y*) for LCP with different focal lengths *f_1_* and *f_2_*, at a working wavelength of 633 nm. Then, by designing nanostructures that act as half‐wave plates, we can then use the propagation phase alongside the geometric phase to create the metasurface. Since the rotation of a structure by an angle *θ* acts in equal and opposite directions for RCP and LCP light, according to PB phase, we can define two equations as follows:

(4) $$\psi_{\mathrm{RCP}}\left(x,y\right)=\phi \left(x,y\right)-2\theta \left(x,y\right)$$

and

(5) $$\psi_{\mathrm{LCP}}\left(x,y\right)=\phi \left(x,y\right)+2\theta \left(x,y\right)$$

Solving these equations leads to:

(6) $$\phi \left(x,y\right)=\frac{\psi_{\mathrm{RCP}}\left(x,y\right)+\psi_{\mathrm{LCP}}\left(x,y\right)}{2}$$

(7) $$2\theta \left(x,y\right)=\frac{\psi_{\mathrm{RCP}}\left(x,y\right)-\psi_{\mathrm{LCP}}\left(x,y\right)}{2}$$

The *ϕ*(*x,y*) term is independent of the rotation of the meta‐atoms, so it can be implemented through propagation phase only, while the *2*θ(*x,y*) term dictates the PB phase contribution. Propagation phase is reliant only on the physical dimensions of the designed nanostructures, while the geometric phase is equal and opposite for each circular polarization of light and depends only on the rotation of the meta‐atom inside the unit cell. This allows for every spatial location on the metalens to contribute to the manipulation of the incident light.

**Figure 2:**
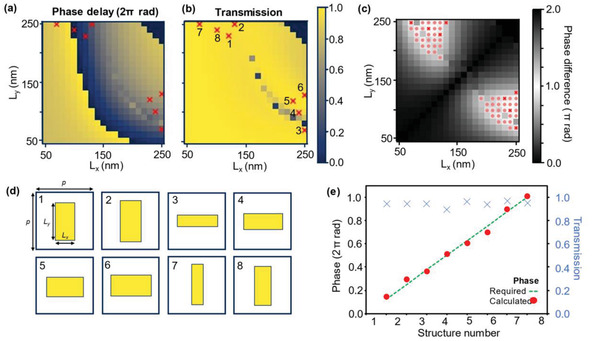
Numerical calculations and design of the individual meta‐atoms. The height (*h*) and the periodicity (*p*) of the meta‐atoms were fixed at *h* = 500 nm and *p =* 350 nm throughout. The calculated a) phase delay and b) total transmission from meta‐atoms with *L_x_* and *L_y_* from 50 to 250 nm under x‐polarized incident light. Note that values of *L_x_^2^* + *L_y_^2^* > *p^2^* are omitted due to the possibility of protruding outside of the unit cell when being rotated to employ the geometric phase. c) The calculated phase difference between x‐ and y‐polarized incident light. Values that satisfy a phase difference of >80% of *π* are denoted by pink circles. d) The selected geometries for the meta‐atoms to cover the required 2*π* range for propagation phase, while also functioning as half‐wave plates. The selected geometries are highlighted with red crosses in (a), (b), and (c), while the numbers in (a) relate to the corresponding geometry. e) The calculated phase (red circles) along with the required phase (green dashed line) for the 8‐step discretization. The calculated phases align well with the required ones, while the transmission (blue crosses) provide a total average transmission of over 95%.

To achieve the required phase profile with meta‐atoms that act as half‐wave plates, a material with a large enough refractive index (*n*) and low extinction coefficient (*k*) is essential. Here, we utilized an optimized low‐loss hydrogenated amorphous silicon (a‐Si:H),^[^ ^41^ ^]^ which exhibits a complex refractive index of 2.88 + 0.00166*i* at 633 nm. The measured refractive index of the material is presented in Note S2, Supporting Information. The almost zero *k* vindicates this choice, moreover the use of silicon allows for the application of mature complementary metal oxide semiconductor (CMOS) processes. To determine the correct geometries of the rectangular nanorods, a parameter sweep was conducted for various values of unit cell periodicity (*p*) and nanostructure height (*h*), alongside the dimensions of the nanostructure in the *x* and *y* directions, while keeping fabrication viability in consideration. This was performed via a homebuilt rigorous coupled‐wave analysis (RCWA) solver. Values of *h* = 500 nm and *p* = 350 nm were determined to best cover the whole range of 2*π* of phase by varying the length of the rectangular nanorods along the x (*L_x_*) and y (*L_y_*) directions, while providing enough flexibility in the meta‐atoms and high transmission. **Figures** **2a** and 2b show the calculated phase delay and total transmission, respectively, for different sized nanorods under x‐polarized incident light. Note that the response to y‐polarized light can be found by simply switching the *x*‐ and *y*‐axis due to symmetry. To satisfy the requirement of the nanorods to act as half‐wave plates, we must select geometries that have a difference of phase retardation equal to *π* between the x‐ and y‐polarization responses. The magnitude of the calculated phase difference between x‐ and y‐polarized light in terms of *π* is shown in Figure 2c. As it is extremely difficult to get a result that is exactly *π*, we allow a leeway of ±20% in the phase difference, that is, 0.8*π* ≤ |ϕ_x –_ *ϕ*_y_| ≤ 1.2*π* to provide more possible options for the dimensions of the nanorods. Comparisons of tightening and loosening these constraints are provided in Note S3, Supporting Information. The geometries that satisfy this relationship are denoted by pink circles in Figure 2c. The choice of height and periodicity allows for a fairly broad range of candidate meta‐atoms, with lengths that are different enough to be feasible for reliable fabrication. Since it is almost impossible to achieve infinitesimally small differences in phase due to the limitations of fabrication and the limited number of meta‐atoms that can be designed inside the unit cell, it is common to discretize the desired phase map and choose structures that produce a continuous gradient from 0 to 2*π*. Here, we choose to discretize the phase into eight equal steps and select the most appropriate structure by minimizing a function as demonstrated in reference.^[^ ^40^ ^]^ We take the maximum error between the required phase at each step and each of the linear polarizations. The function is defined as:

(8) $$\min\{\max\left[\left(T_{\mathrm{mean}}e^{\phi_{\mathrm{required}}}-Te^{\phi_{x}}\right),\;\left(\;T_{\mathrm{mean}}e^{\phi_{\mathrm{required}}+\pi }-Te^{\phi_{y}}\right)\right]\}$$

where *T*_mean_ is the average transmission of all possible geometries, and *T*, *ϕ_x_*, and *ϕ_y_* are the transmission and phase delay for x‐ and y‐polarized light of a given geometry. Visual representations of the selected geometries, the required and calculated phases, and total transmission and are shown in Figures 2d and 2e, respectively. The selected meta‐atoms are also highlighted by red crosses in Figure 2a–c. The properties of the selected nanorods match closely to the required phase profile with a high average transmission of ≈95%, and the phase profiles of the transmitted light for x‐ and y‐polarized light are provided in Note S4, Supporting Information. This allows us to produce a metalens that is both highly efficient and have focusing properties that match closely to the designed parameters.

**Figure 3:**
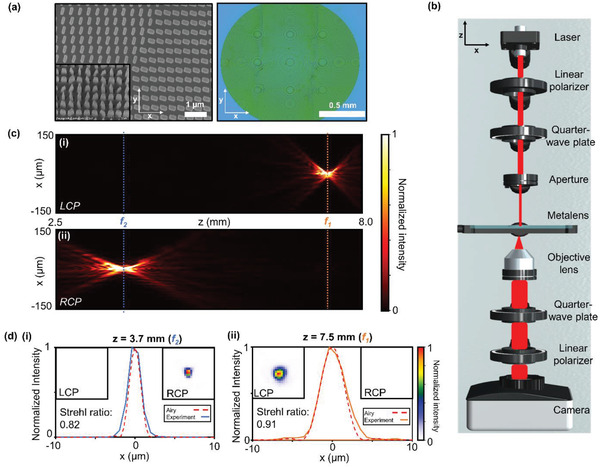
Characterization of the focusing performance of the bifocal metalens. a) (i) Optical microscope and (ii and iii) SEM images of the fabricated bifocal metalens. The diameter of the whole metalens is 1.5 mm. b) Schematic layout of the optical setup. c) The optical field intensity profile along the x‐z plane for i) LCP and ii) RCP incident light. d) The normalized cross‐sectional intensity profiles of the focused light at i) *z* = 7.5 mm (*f*_1_) and ii) *z* = 3.7 mm (*f*_2_). The orange and blue lines denote the locations and measurements of *f*_1_ and *f*_2_, respectively. The insets show 20 × 20 µm *x*–*y* intensity profiles at each position for LCP and RCP incident light. The dashed red lines indicate the shape of the Airy disk of a lens with the same diameter of our metalens. The calculated Strehl ratios are 0.82 and 0.91 for *f*_1_ and *f*_2_, respectively, demonstrating the diffraction limited performance of the bifocal metalens.

The phase profiles of a bifocal metalens with a diameter of 1.5 mm were calculated for focal lengths of *f*_1_ = 7.5 mm, and *f*_2_ = 3.7 mm, which correspond to an NA of 0.1 and 0.2, respectively. The calculated phase profiles are provided in Note S5, Supporting Information. To confirm the focusing ability of the bifocal metalens, the angular spectrum method^[^ ^42^ ^]^ was used to propagate the electric field produced from the metalens in the *z*‐direction. Due to the size of the metalens, it is computationally expensive to store the full field at all points in space, so we cropped the output field to the center 50 pixels that cover a distance of 17.5 µm which is more than enough to capture the key details of the focusing tendency of the metalens. Since the angular spectrum method does not take the circular polarization of the incident light into account, the two focal spots appear consecutively at 3.7 and 7.5 mm as would be expected for linearly polarized light that has components of both LCP and RCP light. These plots are provided in Note S6, Supporting Information. The choice for the diameter of the metalens was limited by fabrication considerations. An optical microscope image and zoomed in scanning electron microscope (SEM) images of the fabricated metalens is shown in **Figure** **3a**. Due to the size of the metalens exceeding the limits of a single exposure of our electron beam lithography process, the fabrication was broken down into smaller sections that were then aligned properly to create the final device. As can be seen in Figure 3a, the metalens was therefore fabricated in twelve sections and some slight variations in the alignment are apparent. However, through the characterization of the metalens, it is confirmed that these alignment errors do not cause any great detriment to the functionality of the device. Furthermore, the SEM images demonstrate that the meta‐atoms were fabricated well.

To characterize the focusing properties of the metalens when illuminated by a 633 nm wavelength laser, we measured the intensity profile in the *x*–*y* focal plane for various propagation distances in the *z*‐direction. A schematic of the optical setup is shown in Figure 3b. The center of the *y* plane was determined by taking the location of the maximum intensity and subsequently, the cross‐sectional images were combined to produce the optical field intensity profile along the *x*–*z* axis as shown for LCP and RCP incidence in Figure 3c. The focusing efficiencies, defined as the ratio of the output power in the focal spot to input power were measured to be 43.5% and 44.0% for LCP and RCP incidence, respectively. A pinhole was used to fit the size of the incident light beam to the diameter of the metalens. The input power is defined as the power of the incident light that passes through the pinhole, linear polarizer, and the quarter‐wave plate, while the output power is defined as the power in the focal spot after passing through the metalens. The measured intensities in the focal planes were normalized and plotted as in Figure 3d along with the diffraction limited Airy disks for a perfect lens with the same diameter as our metalens. The profiles of the focal spots closely resemble those of the perfect Airy disks, implying that the focusing produced by the metalens is also diffraction limited. The insets of Figure 3d show the *x*–*y* field intensities. The calculated Strehl ratios of the focal spots at *f*_1_ and *f*_2_ are 0.82 and 0.91, respectively, highlighting the diffraction limited performance of the bifocal metalens. Furthermore, it is clear to see that there is almost no response for the opposite polarization of light at *f*_1_ and *f*_2_, which is a very important trait for imaging at multiple focal planes without any interference or unwanted noise from the second focal plane.

**Figure 4:**
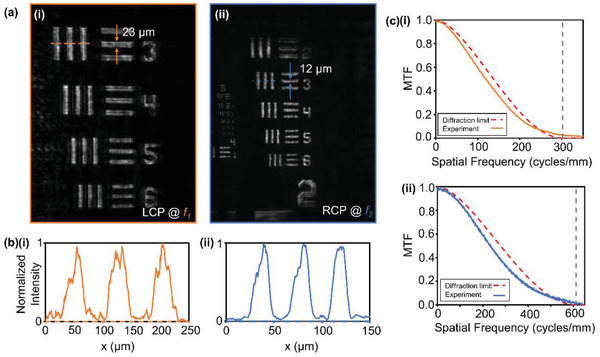
Imaging using the bifocal metalens. a) Captured images of a negative 1951 USAF resolution target using the bifocal metalens for objects in the focal plane of i) *f*_1_ and ii) *f*_2_ with LCP and RCP incident light, respectively. b) Normalized intensity of horizontal cuts across element 3 of group 3 of the target. The location of the cut is shown as a dashed line in (a). c) MTF of the metalens at i) *f*_1_ and ii) *f*_2_.The dashed red lines show the MTF of perfect diffraction limited lenses and the dashed grey lines denote the cutoff frequency.

To prove the imaging capabilities of the metalens, we imaged a negative 1951 USAF Target. Specifically, we highlighted the elements 3 to 6 in group number 3. The results are displayed in **Figure** **4a**. The width of a line in element 3 was measured to be 23 and 12 µm when the object was placed in the focal plane of *f*_1_ and *f*_2_, with LCP and RCP incident light, respectively. Captured images with the opposite polarization can be seen in Note S7, Supporting Information. Plots of the normalized intensity of a horizontal cut across the vertical lines in element 3 are shown in Figure 4b to demonstrate the clear definition between each set of lines. The metalens is able to clearly resolve all target line pairs in group number 3. To uncover the maximum focusing resolution of the metalens we investigated the modulation transfer function (MTF). The MTF describes the spatial frequencies that can be resolved using a lens. The results are shown in Figure 4c. The spatial cutoff frequency quantifies the smallest object that is resolvable, and is defined as:

(9) $$f_{0}=\;\frac{1}{\lambda F_{\#}}$$

where *λ* is the wavelength of light, and *F*_#_ is the focal ratio of the lens, defined as:

(10) $$F_{\#}=\;\frac{f}{D}$$

where *f* is the focal length, and *D* is the diameter of the lens. With our metalens, this gives us cutoff frequencies of 302 and 611 cycles per millimeter for *f*_1_ and *f*_2_, respectively. As can be seen in Figure 4c, the MTF of our metalens closely follows that of the diffraction limited MTF, both reaching the cutoff frequencies. However, both MTFs generally follow below the perfect diffraction limited imaging lines. This can be seen in the aberrations that exist in the captured images. Tests of the resolution performance of our metalens can be found in Note S8, Supporting Information.

**Figure 5:**
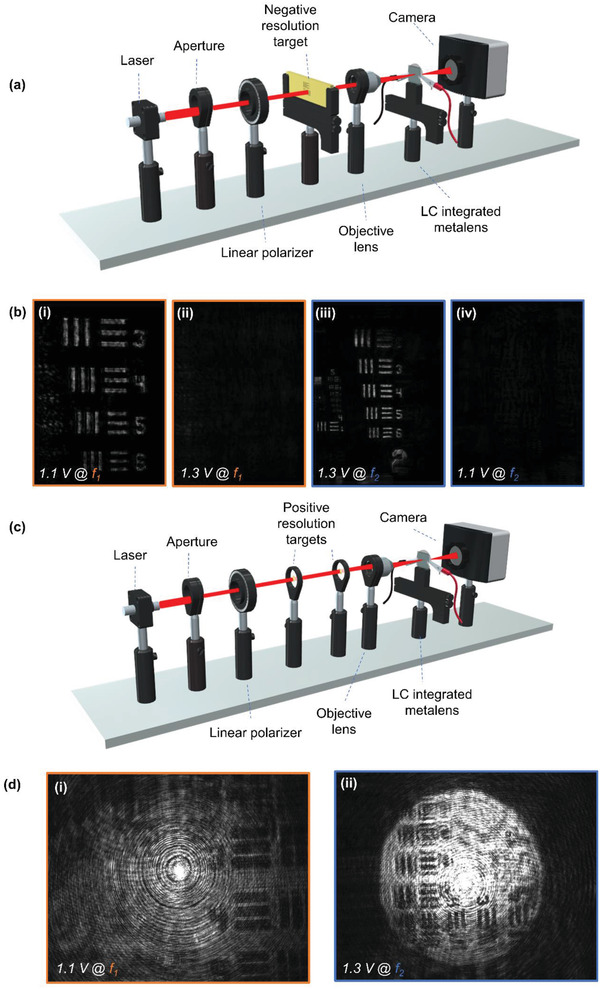
Electrically tunable active imaging using the bifocal metalens combined with LCs. a) Schematic of the optical setup to image the negative 1951 USAF resolution target. b) Captured images of negative 1951 USAF resolution targets at the focal plane of (i) and (ii) *f*_1_, and (iii) and (iv) *f*_2_ with an electrical bias of 1.1 and 1.3 V, to produce LCP and RCP, respectively. c) Schematic of the optical setup to image two positive 1951 USAF resolution targets. d) Captured images of positive 1951 USAF resolution targets at the focal planes of i) *f*_1_ and ii) *f*_2_, with applied electrical biases of 1.1 and 1.3 V, respectively.

To realize an electrically tunable bifocal metalens, an LC cell was combined with the metalens. To precisely control the polarization of the incident light using the LCs, rather than infiltrating the metalens with the LCs^[^ ^32^ ^]^ we chose to combine the LC cell and metalens substrate in a sequential manner. The sandwich‐cell type LC cell consists of 4‐Cyano‐4′‐pentylbiphenyl (5CB) where the LC molecules are tangentially aligned to the substrate along the rubbing direction. As an electric bias is applied to the LC cell, the orientation of the LCs is manipulated, resulting in the desired polarization shift. 45° linearly polarized light was illuminated on the LC‐integrated metalens, then by applying external biases of 1.1 and 1.3 V, LCP and RCP light was produced. A more detailed explanation of the working mechanism is included in Note S9, Supporting Information. As proof‐of‐concept of an electrically tunable bifocal metalens, we demonstrated on and off focusing with a switching speed of around several milliseconds. The same setup was used as previously, but without the need of a quarter‐wave plate to create circularly polarized light, as shown in **Figure** **5a**. Again, we imaged elements 3 to 6 in group number 3 of the negative 1951 USAF Target. The imaging results for the focused and unfocused states are shown in Figure 5b. A video that demonstrates the millisecond switching speed and quality of the imaging can be found in Video S1, Supporting Information. The quality of the imaging is comparable with the static setup using a quarter‐wave plate to produce the circularly polarized light, proving the applicability of the LCs. Furthermore, to demonstrate active switching between multiple targets, we imaged two positive versions of the 1951 USAF Target, rotated at 90° to each other. The imaging results are shown in Figure 5c, and a video of the active focusing can be found in Video S2, Supporting Information. Compared to the negative target images, the quality of the positive target images is somewhere diminished due to the focusing efficiency of the metalens, however the desired images can be clearly seen at the same resolution as the negative target case. The image of the object at *f*_2_ also shows some interference from the object at *f*_1_, which could be attributed to the misalignment of the objects in the focal planes. There is also some evidence of unconverted light that is present in the center of the image that shows as the bright spot. However, the switching of focus between the two distinct objects is clearly demonstrated. We envision that such an electrically tunable metalens will enable rapid zooming and autofocusing functions in cameras, without the requirement of bulky optical components, leading to devices that have much smaller footprints and form factors. This would be extremely useful in compact devices such as smartphones, and could help to further miniaturize augmented (AR) and virtual reality (VR) devices.

## 3 Conclusion

In conclusion, we have numerically designed and experimentally realized a bifocal metalens working at visible wavelengths. We demonstrated tunable diffraction limited focusing at two distinct focal lengths separated by 3.8 mm by applying an external bias onto an LC cell in combination with a metalens. The 1.5 mm diameter metalens produced focusing efficiencies of 43.5% and 44.0% at focal lengths of 7.5 and 3.7 mm, respectively. This high efficiency focusing is achieved through the design of the metasurface to allow every meta‐atom to contribute to the focusing of both LCP and RCP light. Furthermore, imaging with active focusing on a millisecond timescale using the bifocal metalens at the designed wavelength of 633 nm was also experimentally demonstrated. The images of the positive target show some artifacts that we attribute to the imperfect conversion between LCP and RCP light from both the LCs and the metasurface, fabrication defects due to the misalignment of nanostructures on the metalens, as well as imaging alignment difficulties due to the short working focal lengths on the 100 µm scale. Large‐scale printable metasurfaces^[^ ^42^ ^]^ could be a potential solution to overcome the alignment issues when using electron beam lithography. Nevertheless, active switching between the focus of two distinct targets using an electrical bias was experimentally achieved, with switching times in the order of milliseconds. The straightforward combination of metasurfaces and LC has proven a valuable collaboration for creating tunable photonic devices in a simple and effective way. The design strategy demonstrated here could be further expanded to lenses with higher NA and to work at different visible wavelengths with the appropriate optimization of the constituent meta‐atoms, and the possibly of using machine learning techniques^[^ ^43^, ^44^ ^]^ to design more complex metasurfaces could open up the realization of completely focus tunable metalenses. Furthermore, other applications of electrically tunable metasurfaces could be realized, such as controllable beam steering and holography, which could have impact in the fields of LiDAR^[^ ^45^ ^]^ and AR/VR.

## 4 Experimental Section

### Numerical Simulation

An in‐house developed RCWA solver was used to calculate the transmission spectra and phase delay of the meta‐atoms, using x‐polarized incident light.^[^ ^46^ ^]^ Field distributions were calculated using the commercially available FDTD solver Lumerical, from Ansys.

### Metalens

The metalens was fabricated on a glass substrate. A 500 nm thick layer of a‐Si:H was deposited on the substrate using plasma enhanced chemical vapor deposition (PECVD) with gases flow rates of 10 sccm for SiH_4_ and 75 sccm for H_2_. The meta‐atom structures were exposed onto a positive tone photoresist (Microchem, 950 PMMA A2) using a general electron beam lithography process (ELIONIX, ELS‐7800, 80 kV acceleration voltage and 100 pA beam current). The exposed photoresist was developed in methyl isobutyl ketone/Isopropyl alcohol 1:3 solution. Then, a 40 nm thick chromium (Cr) layer was deposited using electron beam evaporation (KVT, KVE‐ENS4004) followed by a lift‐off process. The Cr was used as an etch mask for the a‐Si:H in a dry etching process (DMS, silicon/metal hybrid etcher). The remaining Cr etch mask was removed using Cr etchant (CR‐7).

### LC

The LC modulator fabrication started from the polyimide (Nissan Chemical Korea) coating of glass plates. The polyimides were spin‐coated at 1000 rpm for 10 s and 2500 rpm for 30 s. Then, the plates were baked at 230 °C for 60 min and rubbed to realize a unidirectional alignment of the LCs. The two plates were then assembled with a 10 µm gap, formed with spacer mixed with UV‐glue. The 5CB LCs were injected into the sandwich LC cell. The fabricated LC cell was attached onto the metalens substrate using UV‐glue.

### Focusing

The focal spots of metalens were characterized using a microscope setup. A 633 nm wavelength laser was used to produce a beam of light that was then cropped by an aperture to match the diameter of the metalens. Two mirrors were used to propagate the light at normal incidence. The light was first passed through a linear polarizer (Thorlabs LPVISC050) and then a quarter‐wave plate (Thorlabs AQWP05M‐600) with the axis of the linear polarizer at 45° (or −45°) to the fast axis of the quarter‐wave plate to produce circularly polarized light. The metalens focused the light to the focal points which were imaged by a scanning horizontal microscope using an objective lens (Olympus LMPLFLN‐BD 20x), circularly polarization analyzer, and a CCD camera (Lumenera INFINITY2‐2). A circular‐polarization selective horizontal microscope was mounted on a stage to measure the intensity of the light for a total of 195 measurements at 0.03 mm intervals.

### Imaging

For the imaging of macroscopic objects, the setup was largely that same as the focusing setup. The circularly polarized light was incident on a negative resolution target (Thorlabs R3L1S4N). An objective lens focused the diverging light tightly to ensure that the whole target was incident on the metalens. The resulting images were captured with the same CCD camera. The metalens and CCD camera were mounted on a stage to allow the horizontal modulation of the object‐to‐lens distance.

### Imaging with LC

The LC cell was connected to a function generator (Agilent 33220A) to apply the required bias for electric modulation of the metalens.

## Conflict of Interest

The authors declare no conflict of interest.

## Supporting information

Supporting Information

Supplemental Video 1

Supplemental Video 2

## Data Availability

Data available on request from the authors.
