# Supplementary material for: Electrically Tunable Bifocal Metalens with Diffraction‐Limited Focusing and Imaging at Visible Wavelengths
Source: Adv Sci (Weinh). 2021 Sep 5;8(21):2102646. doi: 10.1002/advs.202102646 (PMC8564427; doi:10.1002/advs.202102646)
Supplement: Supplementary file 1 — Supporting Information [file ADVS-8-2102646-s002.pdf]

WILEY-VCH

© 2021 Wiley-VCH GmbH

ADVANCED SCIENCE Open Access

Supporting Information

for *Adv. Sci.*, DOI: 10.1002/advs.202102646

Electrically Tunable Bifocal Metalens with Diffraction-Limited Focusing and Imaging at Visible Wavelengths

*Trevon Badloe[1†], Inki Kim[1†], Yeseul Kim[1†], Joohoon Kim[1], Junsuk Rho[1,2,3,4]**

Supporting Information

Electrically tunable bifocal metalens with diffraction-limited focusing and imaging at visible wavelengths

*Trevon Badloe[1†], Inki Kim,[1,2†], Yeseul Kim[1†], Joohoon Kim[1], Junsuk Rho[1,3,4,5]**

T. Badloe, Prof. I. Kim, Y. Kim, J. Kim, Prof. J. Rho*
[1]Department of Mechanical Engineering, Pohang University of Science and Technology (POSTECH), Pohang 37673, Republic of Korea
*E-mail: jsrho@postech.ac.kr

Prof. I. Kim
[2]Department of Biophysics, Institute of Quantum Biophysics, Sungkyunkwan University, Suwon 16419, Republic of Korea

Prof. J. Rho
[3]Department of Chemical Engineering, Pohang University of Science and Technology (POSTECH), Pohang 37673, Republic of Korea

Prof. J. Rho
[4]National Institute of Nanomaterials Technology (NINT), Pohang 37673, Republic of Korea

Prof. J. Rho
[5]POSCO-POSTECH-RIST Convergence Research Center for Flat Optics and Metaphotonics, Pohang 37673, Republic of Korea

[†] These authors contributed equally to this work.

Supplementary Note 1: Comparison of experimentally reported varifocal metalenses.

Table S1: Comparison of experimentally reported varifocal metalenses.

**Tuning type**		**Operating wavelength**	**Focal length change**	**Switching speed scale**	**Ref**
Electrical	LC	650 nm	35 µm	Microsecond	[S1]
	Actuation strain	1,550 nm	15 mm	Microsecond	[S2]
	Carrier at Dirac point in graphene	445-630 nm	16.4-26.2 µm	-	[S3]
Thermal	GST phase change material	3.1 µm	500 µm	Nanosecond	[S4]
	VO_2 phase change material	4 µm	200 µm	-	[S5]
	Electro-thermo-optical module (spiral heater)	632 nm	100-150 µm	Millisecond (100 ms)	[S6]
Mechanical	MEMS	915 nm	36 µm	Millisecond	[S7]
	Radially stretched elastic polymer	915 nm	500 µm	Millisecond	[S8]
Electrical	LC	633 nm	3.8 mm	Millisecond	This work

Supplementary Note 2: Measured refractive index of a-Si:H

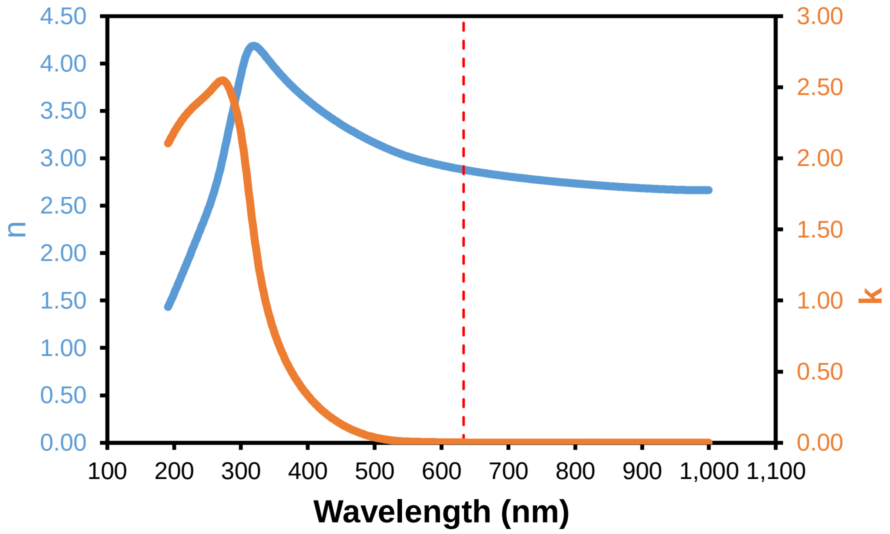

Figure S1: Measured refractive index of a-Si:H. The low-loss a-Si:H has a fairly high refractive index (n), and almost zero extinction coefficient (k) at the working wavelength of 633 nm, denoted by the dashed red line.

The measured refractive index at the working wavelength of 633 nm is 2.88 + 0.00166i. The almost zero extinction coefficient provides a strong platform to design a device that has almost no optical losses due to the material.

Supplementary Note 3: Effect of strictness of π phase difference on the choice of meta-atoms

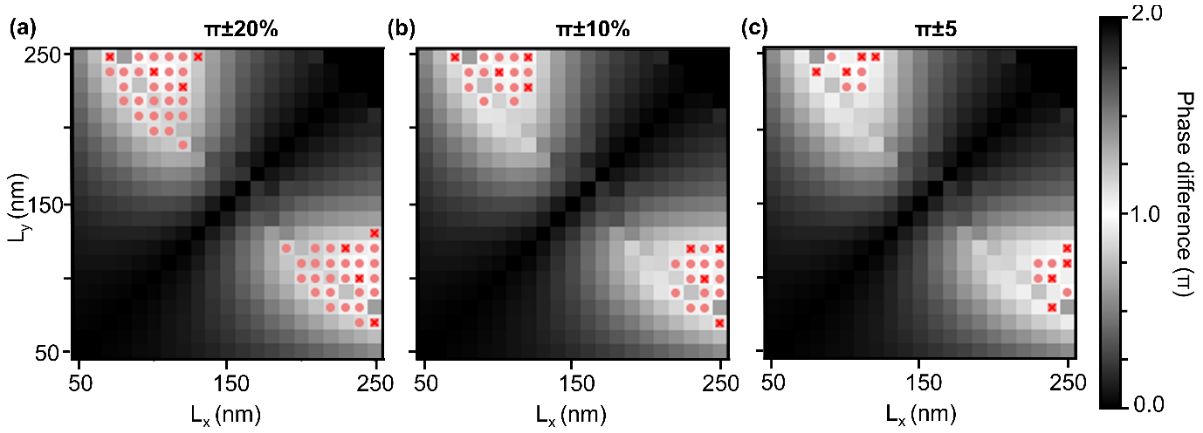

Figure S2: Effect of the strictness of the meta-atoms to act as half-wave plates. For π± (a) 20%, (b) 20%, and (c) 5%.

As the strictness on the requirement of the meta-atoms to act as half-wave plates is increased, the conversion between RCP and LCP is improved. However, this leads to the selection candidates of meta-atoms to realize the required phase retardations to become smaller, limiting the ability to choose meta-atoms that fit the phase gradient profile. Therefore, some trade-off must be undertaken. In addition, when the strictness is increased, as well as the number of candidate structures being reduced (indicated by pink dots in Figure S2), the selected structures (indicated by red crosses in Figure S2) also become closer in terms of their geometric parameters. Since there are limitations to fully control the dimensions of the fabricated meta-atoms down to sub-10 nm accuracy using electron beam lithography, this should be avoided. These problems could be overcome through either a more complex meta-atom design library[9,10] or through computational optimization and design,[11,12] which would increase the up-front computational cost, or through a suitable large-scale fabrication technique that has extreme resolution.[13]

Supplementary Note 4: Phase delay between x- and y-polarized incident light of the selected meta-atoms

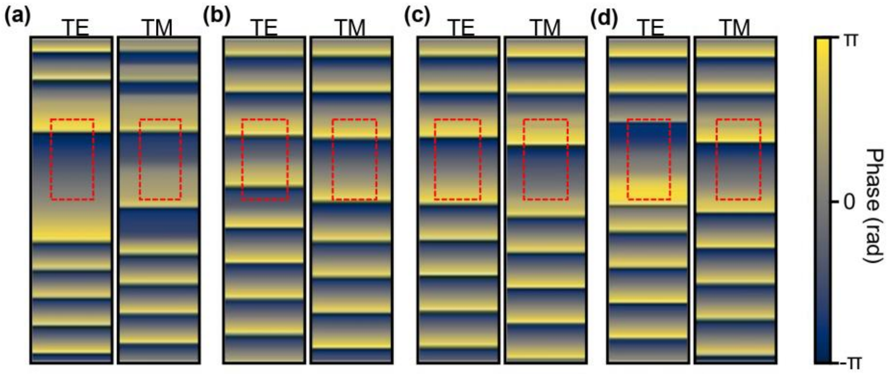

Figure S3: Phase delay between x- and y-polarized incident light. The phase difference between the selected meta-atoms for x- (TE) and y-polarized (TM) incident light is delayed by π. This justifies the choice of the meta-atoms to operate as half-wave plates. (a), (b), (c), and (d) refer to structures 1 and 5, 2 and 6, 3 and 7, and 4 and 8, respectively.

Supplementary Note 5: Phase profiles of the designed metalens

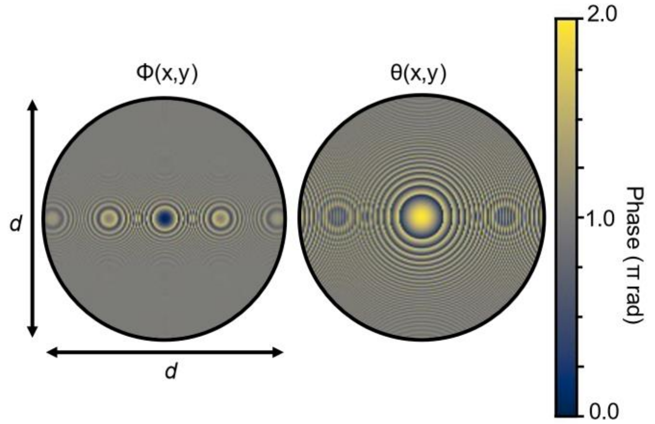

Figure S4: Phase profiles of the bifocal metalens. The phase profiles of the bifocal metalens to produce two focal lengths for different handedness of circular polarization. $\phi(x,y)$ is achieved through the placement of the 8-step propagation phase meta-atoms, while $\theta(x,y)$ is achieved through the rotation of the meta-atoms to produce an extra contribution of geometric phase. Using this encoding method, all elements in the metalens contribute to the focusing of the incident light. The diameter *d* of the metalens is 1.5 mm.

Supplementary Note 6: Simulated performance of the metalens

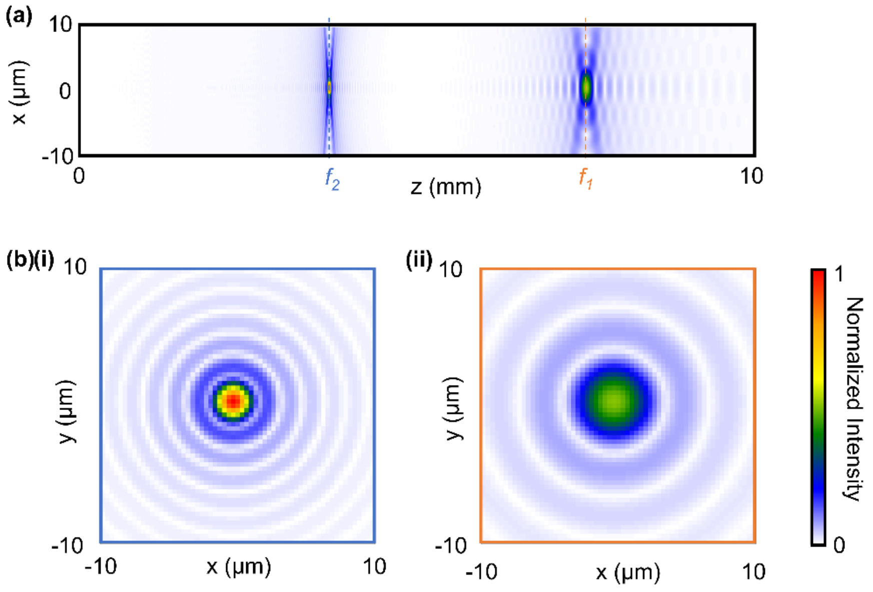

Figure S5: Simulated characterization of the metalens. (a) Optical intensity in the x-z plane, simulated using the angular spectrum method. Two focal spots at the designed focal lengths of 7.5 mm (f_1) and 3.7 mm (f_2) can be clearly seen. (b) The simulated Airy spots of the metalens show the perfect diffraction-limited focusing at (i) f_1 and (ii) f_2. The Airy spots are normalized to the maximum intensity, which is in the focal spot of f_2.

Supplementary Note 7: Imaging with the bifocal metalens

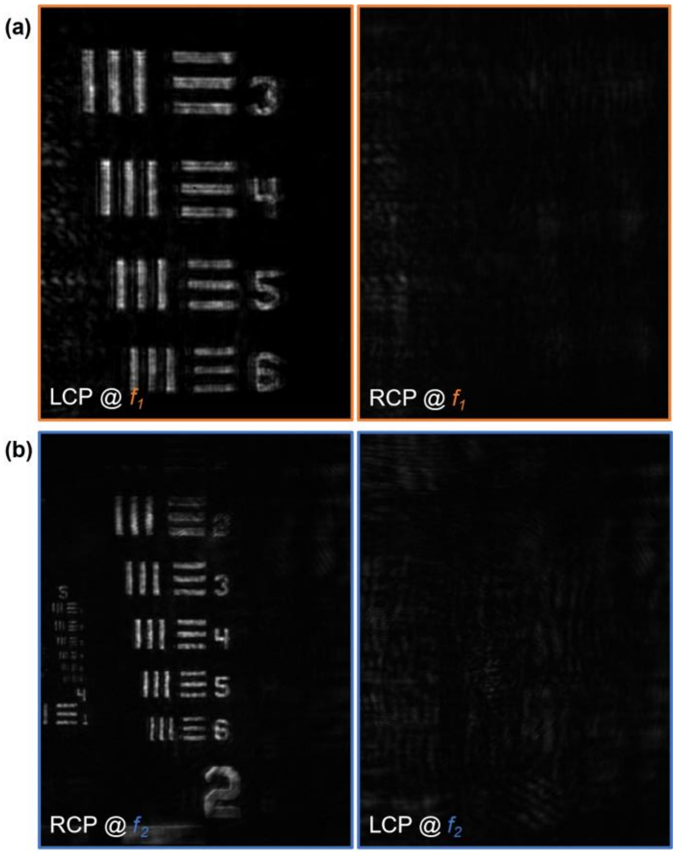

Figure S6: Imaging with the bifocal metalens. Elements 3 to 6 of group 3 of a negative 1951 USAF resolution target were imaged at the focal planes of (a) f_1 and (b) f_2. The object is only imaged when the polarization of the incident light is LCP or RCP for f_1 and f_2, respectively. When the incorrect polarization is incident, an almost completely black image is captured.

Supplementary Note 8: Resolution test of the bifocal metalens

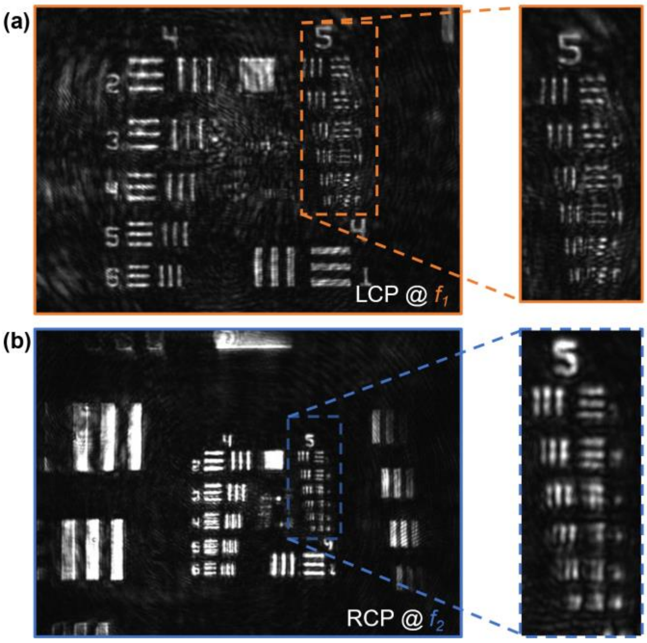

Figure S7: Resolution test of the bifocal metalens. The resolution of the bifocal metalens was tested by imaging group 5 negative 1951 USAF resolution target at the focal plane of (a) f_1 and (b) f_2 with LCP and RCP incidence, respectively. The metalens is able to resolve the three lines up to around element number 3.

Supplementary Note 9: Details of the working mechanism of the LC cell

The specifically designed LC modulator is fabricated on an indium-tin-oxide (ITO) coated glass substrate. A 10 µm thick sandwich-type LC modulator was designed. The LC modulator is composed of 5CB LC molecules that have a birefringence Δn = 0.1834 at 633 nm. Initially, the LCs are oriented parallel to the substrate and the incident beam is 45° linearly polarized (LP 45°) about the rubbing direction. According to the phase retardation τ [rad] which is determined as $\tau = \int_0^d 2\pi\Delta n_{\mathrm{eff}}(z)/\lambda\, dz,$ where d is the thickness of the LC cell, and λ is the wavelength of the transmitted light, the LP 45° light experiences maximum phase retardation of 18.2 rad corresponding to 2.9 turns around a S1 rotation axis on Poincaré sphere (Figure S8a). Once a voltage is applied to the modulator, the LCs start to rotate, and the phase retardation is reduced. By applying the proper electric voltage, LCP or RCP state can be generated (Figure S8b). The polarization state of outgoing light beam from LC cell is measured by Stokes polarization parameters (Figure S8c), so various output polarization states can be achieved. A schematic of the orientation of the rubbing direction of the LCs and the incident polarized light is shown in Figure S8d).

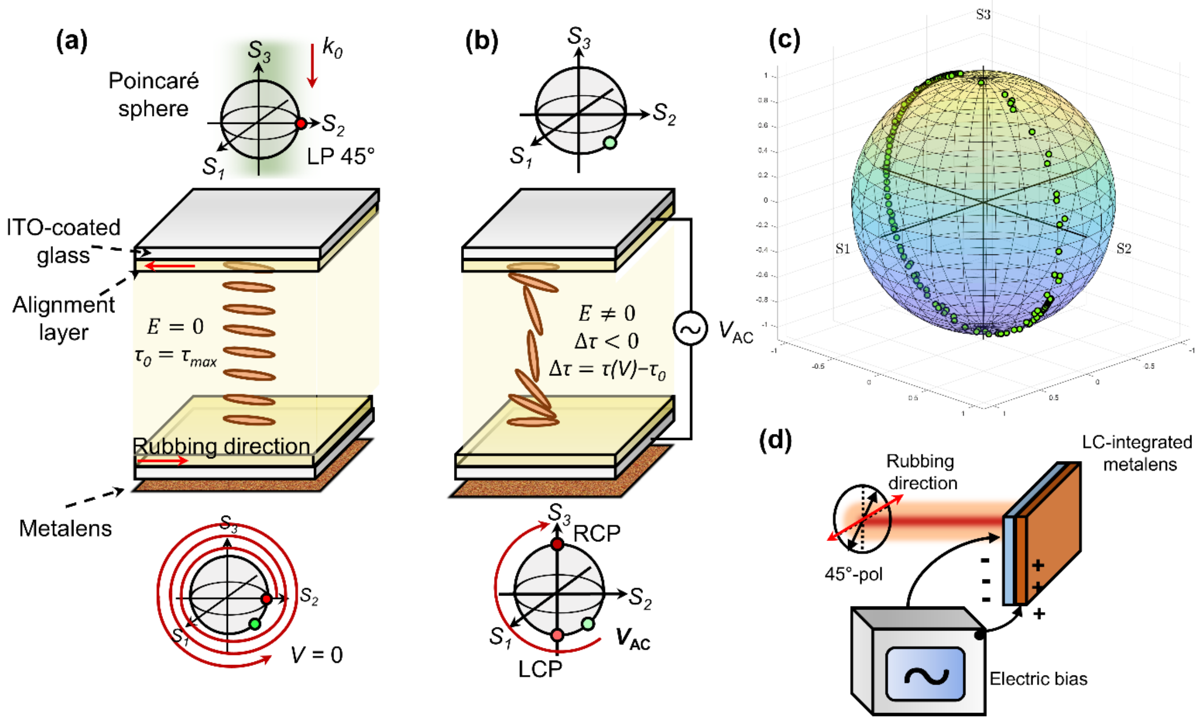

Figure S8: Schematic of the orientation and light manipulation mechanism of the liquid crystals. a) Schematic of the voltage-responsive LCs that are tangentially aligned along the rubbing direction on alignments layers. Without an electric voltage (V = 0), a maximum phase retardation (τ_{max}) is applied on the incoming light resulting in the measurement of 2.9 turns in an anticlockwise direction. b) With a bias V_{AC}, however, the reorientation of LCs occurs parallel to the electric bias direction (perpendicular to the substrate), and thus τ decreases, leading to a change in the polarization of the output light from the incoming 45° linearly polarized light (LP 45°) to the desired polarization state. c) Measured output beam polarization (green points) with respect to V_{AC} on the Poincaré sphere. The green dots indicate the achievable output beam polarizations. d) Schematic of the voltage-responsive bifocal metalens.

References

[S1] C.-Y. Fan, T.-J. Chuang, K.-H. Wu, G.-D. J. Su, *Opt. Express* **2020**, *28*, 10609.

[S2] A. She, S. Zhang, S. Shian, D. R. Clarke, F. Capasso, *Sci. Adv.* **2018**, *4*, eaap9957.

[S3] S. Park, G. Lee, B. Park, Y. Seo, C. bin Park, Y. T. Chun, C. Joo, J. Rho, J. M. Kim, J. Hone, S. C. Jun, *Light Sci. Appl.* **2020**, *9*, 98.

[S4] X. Yin, T. Steinle, L. Huang, T. Taubner, M. Wuttig, T. Zentgraf, H. Giessen, *Light Sci. Appl.* **2017**, *6*, e17016.

[S5] N. Xu, Y. Liang, Y. Hao, M. Mao, J. Guo, H. Liu, H. Meng, F. Wang, Z. Wei, *Nanomaterials* **2020**, *10*, 1135.

[S6] A. Afridi, J. Canet-Ferrer, L. Philippet, J. Osmond, P. Berto, R. Quidant, *ACS Photonics* **2018**, *5*, 4497.

[S7] E. Arbabi, A. Arbabi, S. M. Kamali, Y. Horie, M. Faraji-Dana, A. Faraon, *Nat. Commun.* **2018**, *9*, 812.

[S8] S. M. Kamali, E. Arbabi, A. Arbabi, Y. Horie, A. Faraon, *Laser Photonics Rev.* **2016**, *10*, 1002.

[S9] W. T. Chen, A. Y. Zhu, J. Sisler, Z. Bharwani, F. Capasso, *Nat. Commun.* **2019**, *10*, 1.

[S10] S. Shrestha, A. C. Overvig, M. Lu, A. Stein, N. Yu, *Light Sci. Appl.* **2018**, *7*, 85.

[S11] T. Phan, D. Sell, E. W. Wang, S. Doshay, K. Edee, J. Yang, J. A. Fan, *Light Sci. Appl.* **2019**, *8*, 48.

[S12] D. Zhu, Z. Liu, L. Raju, A. S. Kim, W. Cai, *ACS Nano* **2021**, *15*, 2318.

[S13] D. K. Oh, H. Jeong, J. Kim, Y. Kim, I. Kim, J. G. Ok, J. Rho, *J. Mech. Sci. Technol.* **2021**, *35*, 837.
